# Copper Induces Oxidative Stress and Apoptosis in the Mouse Liver

**DOI:** 10.1155/2020/1359164

**Published:** 2020-01-11

**Authors:** Huan Liu, Hongrui Guo, Zhijie Jian, Hengmin Cui, Jing Fang, Zhicai Zuo, Junliang Deng, Yinglun Li, Xun Wang, Ling Zhao

**Affiliations:** ^1^College of Veterinary Medicine, Sichuan Agricultural University, Wenjiang, Chengdu 611130, China; ^2^Key Laboratory of Animal Diseases and Environmental Hazards of Sichuan Province, Sichuan Agriculture University, Wenjiang, Chengdu 611130, China; ^3^Key Laboratory of Agricultural Information Engineering of Sichuan Province, Sichuan Agriculture University, Yaan, Sichuan 625014, China

## Abstract

Copper (Cu) is an essential trace element involved in the normal physiological processes of animals. However, excessive exposure to Cu can produce numerous detrimental impacts. The aim of this study was to investigate the effects of Cu on oxidative stress and apoptosis as well as their relationship in the mouse liver. Four-week-old ICR mice (*n* = 240) were randomly assigned to different Cu (Cu2+-CuSO4) treatment groups (0, 4, 8, and 16 mg/kg) for periods of 21 and 42 days. The high doses of Cu exposure could induce oxidative stress, by increasing the levels of reactive oxygen species (ROS) and protein carbonyls (PC) and decreasing the activities of antisuperoxide anion (ASA) and antihydroxyl radical (AHR) and content of glutathione (GSH), as well as activities and mRNA expression levels of superoxide dismutase (SOD), catalase (CAT), and glutathione peroxidase (GSH-Px). Moreover, high doses of Cu exposure induced hepatic apoptosis via the mitochondrial apoptotic pathway, as characterized by the depolarization of mitochondrial membrane potential (MMP); significantly increased mRNA and protein expression levels of cytosolic cytochrome (Cyt c), apoptosis-inducing factor (AIF), endonuclease G (Endo G), apoptosis protease-activating factor-1 (Apaf-1), cleaved caspase-9, cleaved caspase-3, cleaved PARP, Bcl-2 antagonist killer (Bak), Bcl-2-associated X protein (Bax), and Bcl-2-interacting mediator of cell death (Bim); and decreased mRNA and protein expression levels of B-cell lymphoma-2 (Bcl-2) and Bcl-extra-large (Bcl-xL). Furthermore, the activation of the tumor necrosis factor receptor-1 (TNF-R1) signaling pathway was involved in Cu-induced apoptosis, as characterized by the significantly increased mRNA and protein expression levels of TNF-R1, Fas-associated death domain (FADD), TNFR-associated death domain (TRADD), and cleaved caspase-8. These results indicated that exposure to excess Cu could cause oxidative stress triggered by ROS overproduction and diminished antioxidant function, which in turn promoted hepatic apoptosis via mitochondrial apoptosis and that the TNF-R1 signaling pathway was also involved in the Cu-induced apoptosis.

## 1. Introduction

Copper (Cu) is an essential trace element involved in the normal physiological processes of animals [1]. Despite its necessity for various metabolic processes and enzyme activities [2], chronic overexposure to Cu may produce some detrimental effects on our body. Generally, occupational exposure to Cu can result in Cu toxicity among industrial workers [3]. In animals, long-term intake of Cu compounds from different origins represents the most common form of Cu poisoning. The metabolism of Cu is mainly regulated by the liver, where it can be released into the circulatory system or excreted via the bile [1]. During chronic Cu toxicity, Cu is gradually accumulated in the liver without producing any obvious signs or symptoms. When the hepatic Cu storage capacity is exceeded, it may result in hepatocellular lesions, and consequently, the liberation of Cu from the liver into the blood stream triggers hemolysis, jaundice, and renal insufficiency [4].

Our previous studies have indicated that excessive Cu exposure can induce oxidative stress in the brain [5, 6] and spleen [7] in chicken, reduce the activities of copper-zinc superoxide dismutase (CuZn-SOD) and glutathione peroxidase (GSH-Px), and increase the contents of malondialdehyde (MDA) and hydroxyl radical in the liver [8, 9] and kidney [10] of ducklings. Oxidative stress is considered to reflect an imbalance between the production of reactive oxygen species (ROS) and the ability of the body to detoxify this intermediate [11]. The overproduction of ROS affects mainly biomembranous unsaturated fatty acids and decreases membrane fluidity and disrupts membrane structure and function [12]. The findings from *in vitro* and *in vivo* studies have demonstrated that Cu possesses the capacity to initiate oxidative damage [13–17]. Ozcelik and coworkers [18] have also found that excess Cu exposure can induce oxidative stress and suppress the antioxidant defense system in the rat liver. However, much less is known about the exact mechanism of Cu-induced oxidative stress in the liver.

It has been widely accepted that oxidative stress is an apoptotic inducer. Apoptosis, or programmed cell death, is a naturally occurring cell death process, which is responsible for the normal development and homeostasis in all multicellular organisms [19]. Cu-induced apoptosis has been reported in vivo [20]. As an intrinsic apoptosis pathway, the mitochondrial apoptosis pathway plays a key role in cell death. The key members in this pathway include B-cell lymphoma-2 (Bcl-2) family protein, mitochondrial proapoptosis proteins, and caspases. Several studies have demonstrated that Cu induces apoptosis in the liver via increasing the protein expression levels of caspase-3, caspase-8, caspase-9, and Bcl-2-associated X protein (Bax) [20, 21]. It has been found that overexposure to Cu can result in mitochondrial dysfunction and increase the protein expression levels of cytosolic cytochrome (Cyt c) in the chicken liver [22], mouse brain [23], and chicken testis [24]. Besides, the death receptor pathway is also one of the main apoptosis signaling pathways [25], which belongs to the extrinsic apoptosis pathway. At the cell surface level, some of the well-characterized death receptors include Fas receptor and tumor necrosis factor receptor-1 (TNF-R1) that can bind to tumor necrosis factor alpha (TNF-*α*), a proinflammatory cytokine. Binding of TNF-*α* to TNF-R1 usually results in caspase activation and apoptosis [26]. At present, there are no reports about the relationship between Cu-induced hepatic apoptosis and death receptor pathway. Also, the *in vivo* role of the mitochondrial apoptotic pathway in Cu-induced hepatic apoptosis has not been reported thus far.

Based on the above findings, excess Cu intake can induce oxidative stress and apoptosis, but the underlying mechanisms remain unclear. There has been little systematic research focused on the relationship between oxidative stress and apoptosis in the mouse liver with excessive copper exposure. Hence, the present study was aimed at elucidating the possible molecular mechanism underlying Cu-induced oxidative stress and apoptosis as well as their relationship in the mouse liver.

## 2. Materials and Methods

### 2.1. Chemicals

Cu sulfate was purchased from Chengdu Kelong Chemical Co., Ltd. (Chengdu, China). ROS Assay Kit (88-5930-74) was obtained from Thermo Fisher, China. Reagent kits for the determination of biochemical parameters (protein carbonyls (PC), A087-2; MDA, A003-1; antihydroxyl radical (AHR), A018; antisuperoxide anion (ASA), A052; catalase (CAT), A007-1; glutathione reductase (GR), A062; glutathione (GSH), A006-2; GSH-Px, A005-a; glutathione-s-transferase (GST), A004; and superoxide dismutase (SOD), A001-1) were purchased from the Nanjing Jiancheng Bioengineering Institute of China (Nanjing, China). PrimeScript™ RT Reagent Kit (cat no. RR047A), SYBR® Premix Ex Taq™ II (cat no. RR820A), and RNAiso Plus (cat no. 9109) were supplied by Takara Biotechnology Co., Ltd. (Dalian, Liaoning, China). BD Pharmingen™ PE Annexin V Apoptosis Detection Kit I (cat no. 559763) and Mitochondrial Membrane Potential Detection JC-1 Kit (cat no. 551302) were obtained from BD Bioscience (San Jose, CA, USA). Radioimmunoprecipitation assay (RIPA) lysis buffer (cat no. P0013C), phenylmethylsulfonyl fluoride (PMSF; cat no. ST506), tissue mitochondria isolation kit (cat no. C3606), and bicinchoninic acid (BCA) protein assay kit (cat no. P0012) were obtained from Beyotime Biotechnology (Nanjing, China). Mouse Cyt c (cat no. ab133504), endonuclease G (Endo G; cat no. ab76122), apoptosis-inducing factor (AIF; cat no. ab32516), Fas-associated death domain (FADD; cat no. ab124812), and TNFR-associated death domain (TRADD; cat no. ab110644) were purchased from Abcam (Cambridge, UK). Apoptosis protease-activating factor-1 (Apaf-1; cat no. 8723T), Bax (cat no. 2772), Bcl-2 antagonist killer (Bak; cat no. 12105), Bcl-2-interacting mediator of cell death (Bim; cat no. 2933), Bcl-2 (cat no. 2870), Bcl-extra-large (Bcl-xL; cat no. 2764), cleaved caspase-3 (cat no. 9664), cleaved caspase-9 (cat no. 9509), cleaved PARP (cat no. 5625), TNF-R1 (cat no. 13377S), cleaved caspase-8 (cat no. 8592T), anti-rabbit IgG (cat no. 7074), *β*-actin (cat no. 4970), and SDHA (cat no. 5839S) were obtained from Cell Signaling Technology (Danvers, MA, USA).

### 2.2. Animals and Treatments

Four-week-old healthy ICR mice (*n* = 240, half male and female) were purchased from the Experimental Animal Corporation of Dossy in Chengdu, China. Food and water were provided ad libitum. After 1 week of acclimatization, the mice were randomly divided into four different groups (*n* = 60). The control group received intragastric doses of distilled water, while the experimental groups received intragastric doses of 4, 8, or 16 mg/kg Cu (Cu^2+^-CuSO4; Chengdu Kelong Chemical Co., Ltd., Chengdu, China). Prior to the experiments, Cu sulfate was diluted with distilled water. The gavage doses for all the four groups were 1 mL per 100 g body weight once daily for 42 days. After 21 and 42 days of treatment, all mice were humanely killed and liver samples were collected from sacrificed mice for subsequent analyses.

The use of mice and all experimental procedures were approved by the Animal Care and Use Committee of Sichuan Agricultural University (Chengdu, China).

### 2.3. Detection of the Hepatic Growth Index

After body weights were recorded, eight mice per group were humanely killed on days 21 and 42 of the experiment. Weights were recorded for each liver. The liver growth index (GI) was calculated according to the following formula:

(1) $$\begin{array}{c} \text{GI}=\frac{\text{organ weight }\left(\text{mg}\right)}{\text{body weight }\left(\text{g}\right)}. \end{array}$$

### 2.4. Detection of Hepatic Cu Concentration

0.5 g portion of the liver samples (*n* = 8 per group, half male and female) was wet-digested with 9 mL HNO_3_ and 1 mL HClO_4_ (Chengdu Kelong Chemical Co., Ltd., China) for 24 h in the tube that can withstand high temperature. These tubes were then placed on a hot plate until the liquid was evaporated and the residue was suspended with 10 mL 0.1 M/L HNO_3_. Samples were then analyzed for Cu with a flame atomic absorption spectrophotometer (AAS700, PerkinElmer, USA).

### 2.5. Pathological Assessment

The liver samples (*n* = 8 per group, half male and female) were fixed in 4% paraformaldehyde solution, dehydrated in ethanol, and embedded in paraffin. Then, serial slices at 4 *μ*m thickness were prepared and stained with hematoxylin and eosin (HE). Finally, the stained slices were subjected to histopathological examination using a light microscope.

### 2.6. Detection of Hepatocellular ROS Production by Flow Cytometry

The liver samples (*n* = 8 per group, half male and female) were prepared immediately for the measurement of ROS levels by flow cytometry. Briefly, the liver samples were crushed, filtered through a 350-mesh nylon membrane, centrifuged at 600 × *g* for 5 min, and adjusted to a cell density of 1.0 × 10^6^ cells/mL with phosphate-buffered saline (PBS). Approximately 300 *μ*L of cell suspension was collected, transferred into a new centrifuge tube, and stained with 2′-7′dichlorofluorescin diacetate (DCFH-DA; 10 *μ*M) for 20 min at 37°C. Subsequently, the cells were washed with PBS and centrifuged again at 600 × *g* for 5 min. After discarding the supernatant, the cells were resuspended in 0.5 mL PBS and counted with a BD FACSCalibur flow cytometer.

### 2.7. Measurement of Oxidative and Antioxidant Parameters in the Liver

The liver samples (*n* = 8 per group, half male and female) were rinsed in chilled saline solution, weighed and homogenized in nine volumes of ice-cold 0.9% NaCl solution, and centrifuged at 3500 rpm for 10 min at 4°C. The supernatants were collected for detecting the activities of CAT, GR, SOD, GST, GSH-Px, ASA, and AHR, as well as contents of GSH, MDA, and PC in the liver by biochemical methods according to the instructions of the reagent kits [27].

### 2.8. Detection of mRNA Expression Levels by qRT-PCR

The liver samples (*n* = 8 per group, half male and female) were stored in liquid nitrogen and homogenized for RNA extraction. The method of RNA extraction was the same as that described by Lu and coworkers [25]. The total RNA of the liver samples was extracted using RNAiso Plus (9109; Takara, China). The cDNA was synthesized using PrimeScript™ RT Reagent Kit (RR047A; Takara, China) according to the manufacturer's instructions. The cDNA sequences of CAT, GR, GSH-Px, GST, CuZn-SOD, manganese superoxide dismutase (MnSOD), Cyt c, Endo G, AIF, Apaf-1, caspase-3, caspase-8, caspase-9, PARP, Bcl-2, Bax, Bak, Bim, Bcl-xL, TNF-R1, FADD, and TRADD were obtained from the NCBI database. *β*-Actin was used as a reference gene. The primers (see Table 1) were designed and synthesized by Sangon Biotech (Shanghai, China).

qRT-PCR amplification was performed on a Model C1000 Thermal Cycler (Bio-Rad, USA) using the SYBR® Premix Ex Taq™ II system (RR820A; Takara, China) according to the standard protocols. The relative expression level of each target gene was analyzed by the 2^−ΔΔCT^ method.

### 2.9. Evaluation of Hepatocyte Apoptosis by Flow Cytometry

The liver samples (*n* = 8 per group, half male and female) were ground to form a cell suspension that was filtered through a 350-mesh nylon screen. The cells were washed twice with cold PBS (phosphate buffer solution; pH 7.2-7.4) and then suspended in PBS to a final concentration of 1 × 10^6^ cells/mL. Approximately 100 *μ*L of the cell suspension was transferred into a 5 mL culture tube and stained with PE Annexin V and 7-aminoactinomycin (7-AAD). The mixture was gently vibrated and incubated for 15 min in the dark. Then, 400 *μ*L of 1× binding buffer was added into each tube. Finally, the rates of hepatic apoptosis were analyzed by the BD FACSCalibur flow cytometer.

### 2.10. Analysis of Mitochondrial Membrane Potential (MMP) in Hepatocytes by Flow Cytometry

The liver samples (*n* = 8 per group, half male and female) were ground to form a cell suspension and then filtered through a 350-mesh nylon screen. The cells were washed twice with ice-cold PBS (pH 7.2-7.4) and suspended in PBS to a final concentration of 1 × 10^6^ cells/mL. Approximately 100 *μ*L of cell suspension was transferred into a 5 mL tube and then incubated with JC-1 (5,5′,6,6′-tetra-chloro-1,1′,3,3′-tetraethylbenzimidazolyl-carbocyanineiodide) working solution for 15 min at 37°C in a 5% CO_2_ incubator. The staining solution was removed, and the cells were washed twice with JC-1 staining buffer. Finally, MMP (*ΔΨ*m) was measured by the BD FACSCalibur flow cytometer within 30 min.

### 2.11. Detection of Protein Expression Levels by Western Blotting

Mitochondrial cytosolic protein was extracted according to the specification of a tissue mitochondria isolation kit. Total protein was extracted with the RIPA lysis buffer, and its concentrations were quantitated by a BCA protein assay kit. Protein samples were separated by 10%-15% sodium dodecyl sulfate-polyacrylamide gel electrophoresis (SDS-PAGE) and transferred onto nitrocellulose filter membranes. The membranes were then blocked with 5% fat-free milk for 1 h, followed by overnight incubation with primary antibodies at 4°C. The primary antibodies were Cyt c, AIF, Endo G, Apaf-1, cleaved caspase-3, cleaved caspase-9, cleaved caspase-8, cleaved PARP, Bax, Bak, Bim, Bcl-2, Bcl-xl, TNF-R1, FADD, and TRADD. After washing with PBST (PBS-Tween), the membranes were incubated with biotin-conjugated secondary antibodies for 1 h and then washed again with PBST. Finally, the protein blots were visualized by ECL™ (Bio-Rad, Hercules, CA, USA) and X-ray film. The data of protein expression were analyzed using ImageJ2x software.

### 2.12. Statistical Analysis

To analyze the data, one-way analysis of variance (ANOVA) was carried out by using SPSS software version 18.0. All results were expressed as mean ± standard deviation. *p* < 0.05 was referred to as significant, and *p* < 0.01 was considered highly significant.

## 3. Results

### 3.1. Changes of Body Weight, Hepatic Cu Concentration, and Hepatic Growth Index

**Figure 1:**
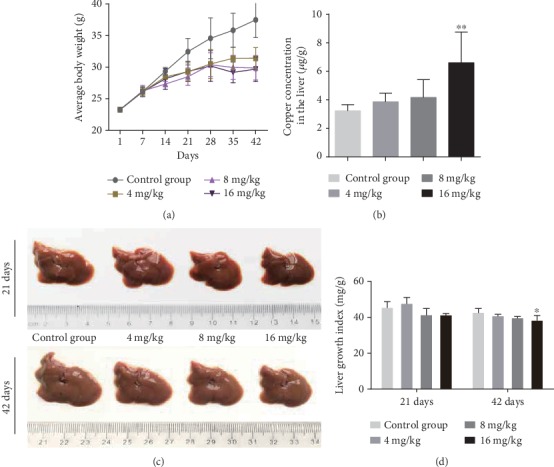


Cu, as an important trace element, is helpful for animal growth in an appropriate level. However, excessive intake of Cu may produce some detrimental effects on bodies. As shown in Figure 1(a), the mice in the 4, 8, and 16 mg/kg Cu treatment groups grew much slower than those in the control group.

The levels of Cu accumulation in the liver were assessed at 42 days of the experiment. The result showed that Cu concentration in the liver was significantly higher (*p* < 0.01) in the 16 mg/kg Cu treatment group than in the control group at day 42 of the experiment (Figure 1(b)).

Hepatic development is evaluated based on the hepatic growth index (GI) values. GI values were decreased (*p* < 0.05) in the 16 mg/kg Cu treatment group at day 42 of the experiment when compared to the control group (Figure 1(d)).

### 3.2. Histopathological Lesions in the Liver

**Figure 2:**
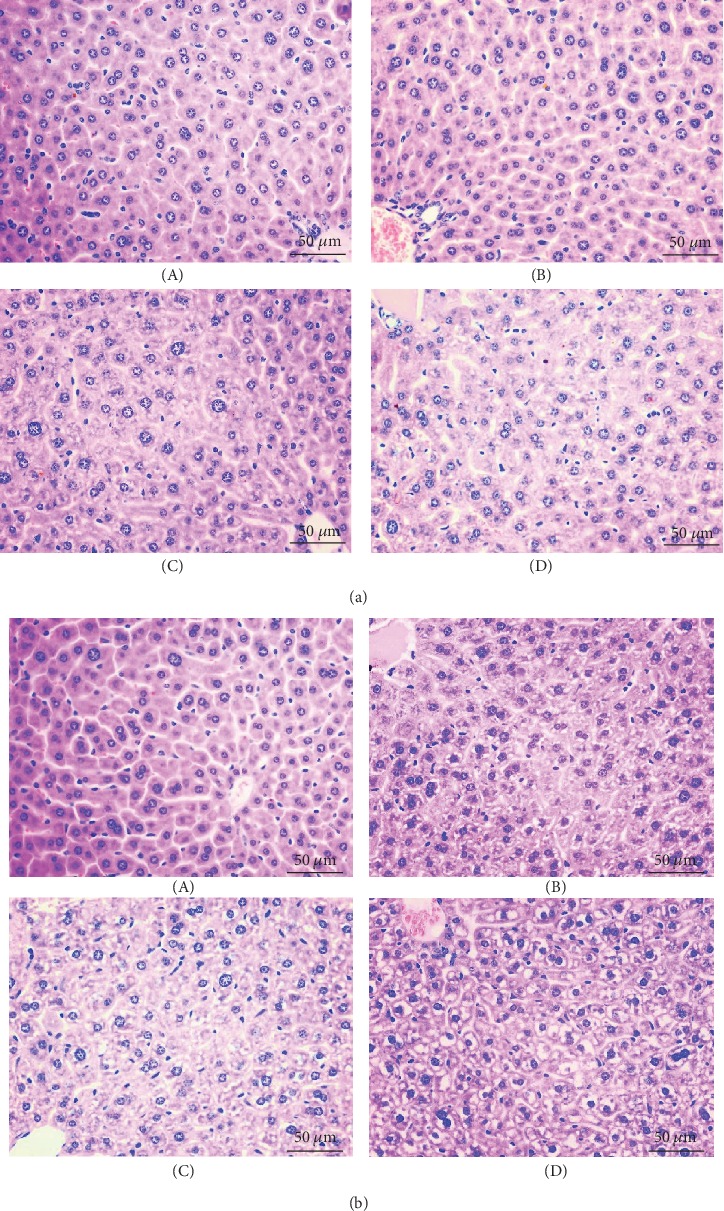


As shown in Figure 2, the number of hepatic cells with granular and vacuolar degeneration was increased by Cu exposure in a dose- and time-dependent manner. Tiny particles as well as small vacuoles could be found in the cytoplasm of these degenerated hepatocytes. Karyorrhexis, karyolysis, and hypochromatosis were observed in the necrotic hepatocytes, especially in the 8 and 16 mg/kg Cu treatment groups. However, the above lesions were not observed in the control group.

### 3.3. Oxidative Stress Levels in the Liver

**Figure 3:**
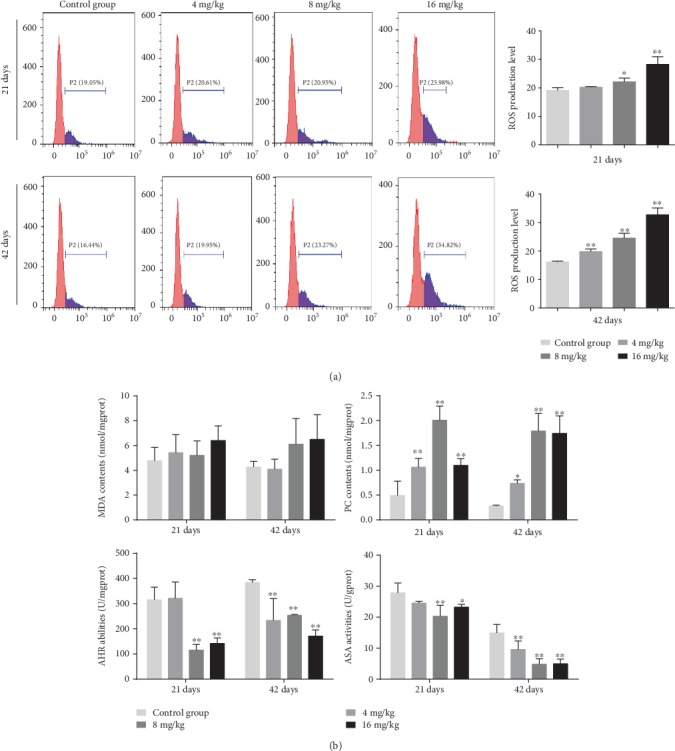


As shown in Figures 3(a) and 3(b), the levels of ROS production were increased (*p* < 0.05 or <0.01) in the 8 and 16 mg/kg Cu treatment groups at days 21 and 42 and in the 4 mg/kg Cu treatment group at day 42 when compared to the control group.

In addition, the contents of PC were increased (*p* < 0.05 or <0.01) in the three Cu treatment groups at days 21 and 42 (Figure 3(c)). However, MDA contents did not differ among all the four groups (Figure 3(c)). Besides, the abilities of ASA and AHR were decreased (*p* < 0.05 or <0.01) in the 8 and 16 mg/kg Cu treatment groups at days 21 and 42 and in the 4 mg/kg Cu treatment group at day 42 as compared to the control group.

### 3.4. Antioxidant Enzyme Activities and GSH Contents in the Liver

**Figure 4:**
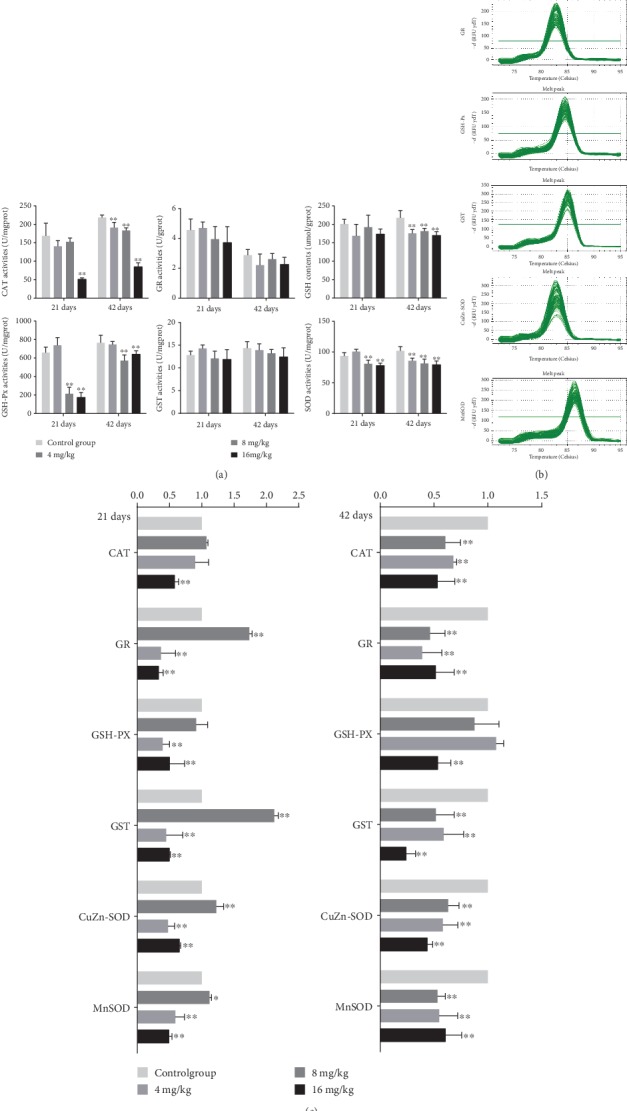


The activities of CAT were significantly decreased (*p* < 0.01) in the 16 mg/kg Cu treatment group at days 21 and 42 as well as in the 4 and 8 mg/kg Cu treatment groups at day 42 when compared to the control group (Figure 4(a)). Besides, the activities of GSH-Px were markedly lower (*p* < 0.01) in the 8 and 16 mg/kg Cu treatment groups than in the control group at days 21 and 42. Similarly, the activities of SOD were significantly decreased (*p* < 0.01) in the 8 and 16 mg/kg Cu treatment groups at days 21 and 42 as well as in the 4 mg/kg Cu treatment group at day 42. Likewise, the contents of GSH were decreased (*p* < 0.01) in the three Cu treatment groups at day 42 when compared with the control group. However, the activities of GR and GST were not significantly changed between the four groups. All results can be found in Figure 4(a).

### 3.5. The mRNA Expression Levels of Antioxidant Enzymes in the Liver

The melting curves of CAT, GR, GSH-Px, GST, CuZn-SOD, and MnSOD are presented in Figure 4(b), and there was only one peak for each PCR product. Compared with the control group, the mRNA expression levels of CAT were significantly lower (*p* < 0.01) in the 16 mg/kg Cu treatment group at days 21 and 42 as well as in the 4 and 8 mg/kg Cu treatment groups at day 42 (Figure 4(c)). The mRNA expression levels of GSH-Px were markedly decreased (*p* < 0.01) in the 16 mg/kg Cu treatment group at days 21 and 42 as well as in the 8 mg/kg Cu treatment group at day 21. The mRNA expression levels of GR, GST, CuZn-SOD, and MnSOD were reduced (*p* < 0.05 or <0.01) in the 8 and 16 mg/kg Cu treatment groups at days 21 and 42 as well as in the 4 mg/kg Cu treatment group at day 42. On the contrary, the mRNA expression levels of these genes in the 4 mg/kg Cu treatment group at day 21 were higher (*p* < 0.05 or <0.01) than those in the control group (Figure 4(c)).

### 3.6. Apoptotic Rates and Depolarization of Mitochondrial Membrane Potential (MMP) in the Liver

**Figure 5:**
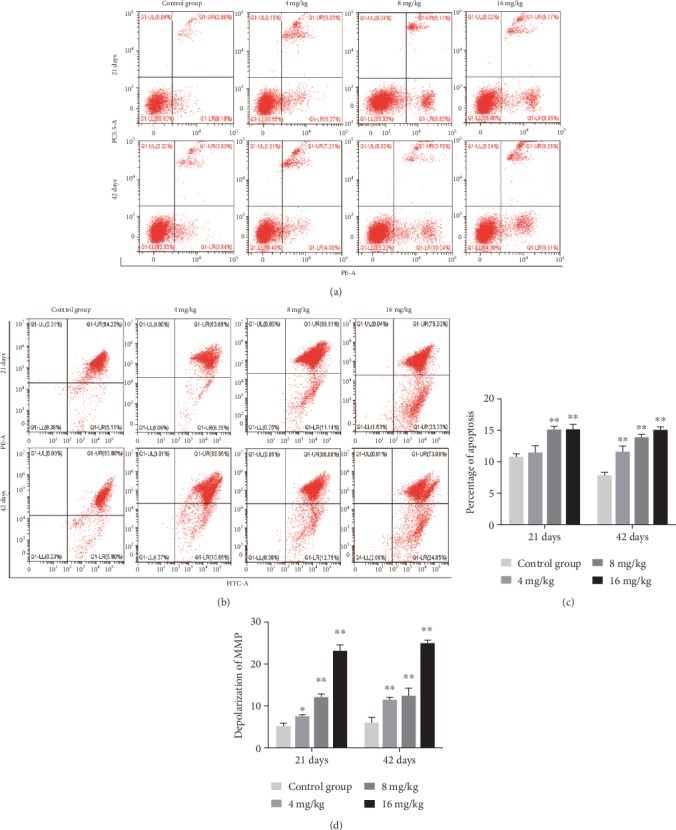
Cu induced apoptosis and depolarization of MMP in the liver at 21 and 42 days of experiment. (a) Apoptosis analyzed by flow cytometry. (b) Depolarization of MMP analyzed by flow cytometry. (c) Quantitation of percentage of total apoptotic cells. (d) Quantitation of depolarization of MMP. Data are presented as the means ± standard deviation (*n* = 8). ^∗^*p* < 0.05 and ^∗∗^*p* < 0.01, compared with the control group.

The percentages of hepatic apoptosis were significantly higher (*p* < 0.01) in the 8 and 16 mg/kg Cu treatment groups at days 21 and 42 as well as in the 4 mg/kg Cu treatment group at day 42 than in the control group (Figures 5(a) and 5(c)).

As shown in Figures 5(b) and 5(d), the proportions of hepatocytes depolarized with MMP collapse were significantly increased (*p* < 0.05 or <0.01) in the three Cu treatment groups at days 21 and 42 when compared to the control group.

### 3.7. Changes in the Protein and mRNA Expression Levels of Mitochondrial Apoptosis-Related Parameters in the Liver

**Figure 6:**
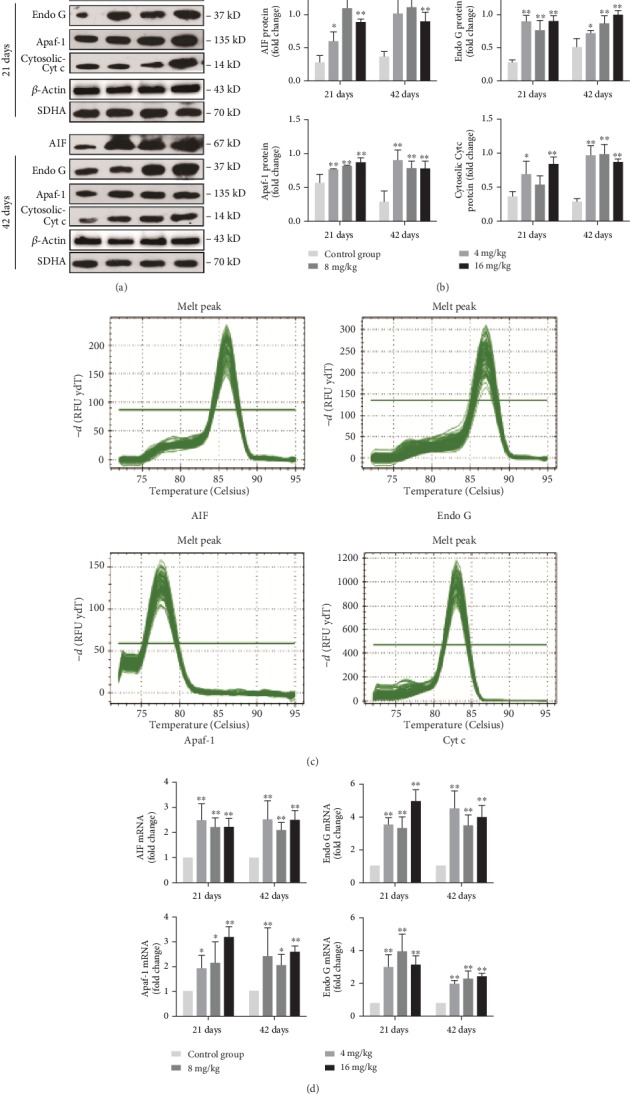
Changes of protein and mRNA expression levels of AIF, Endo G, Apaf-1, and Cyt c in the liver at 21 and 42 days of experiment. (a) The western blot assay of AIF, Endo G, Apaf-1, and Cyt c. (b) The relative protein expression levels of AIF, Endo G, Apaf-1, and Cyt c. (c) The melting curve analysis of AIF, Endo G, Apaf-1, and Cyt c. (d) The relative mRNA expression levels of AIF, Endo G, Apaf-1, and Cyt c. Data are presented as the means ± standard deviation (*n* = 8). ^∗^*p* < 0.05 and ^∗∗^*p* < 0.01, compared with the control group.

As presented in Figures 6(a) and 6(b), the protein levels of AIF, Endo G, and Apaf-1 in the three Cu treatment groups at days 21 and 42 were significantly higher (*p* < 0.05 or <0.01) than those in the control group. The protein levels of cytosolic Cyt c were increased (*p* < 0.05 or <0.01) in the 4 and 16 mg/kg Cu treatment groups at days 21 and 42 as well as in the 8 mg/kg Cu treatment group at day 42 when compared to the control group. Consistent with these protein expression data, the mRNA expression levels of AIF, Endo G, Apaf-1, and Cyt c were upregulated (*p* < 0.05 or <0.01) in the three Cu treatment groups at days 21 and 42 when compared to the control group (Figures 6(c) and 6(d)).

**Figure 7:**
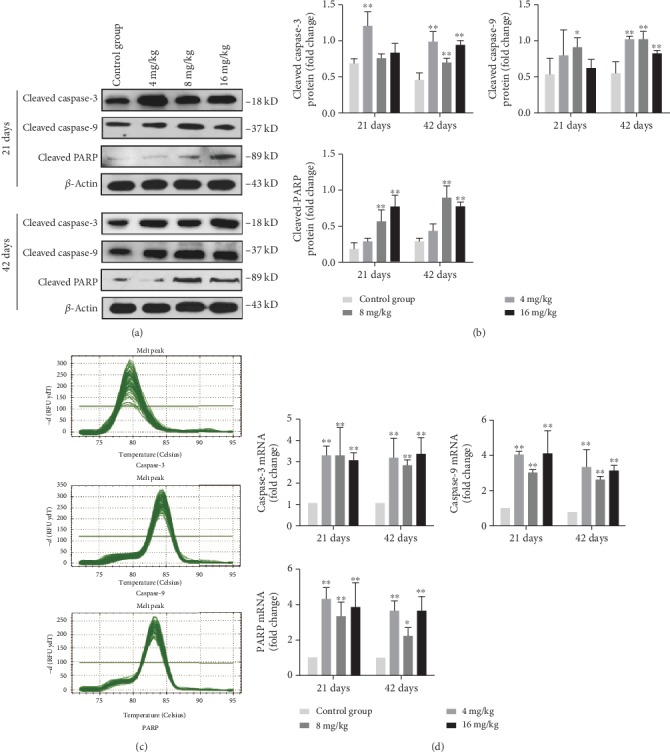
Changes of protein and mRNA expression levels of caspase-3, caspase-9, and PARP in the liver at 21 and 42 days of experiment. (a) The western blot assay of cleaved caspase-3, cleaved caspase-9, and cleaved PARP. (b) The relative protein expression levels of cleaved caspase-3, cleaved caspase-9, and cleaved PARP. (c) The melting curve analysis of caspase-3, caspase-9, and PARP. (d) The relative mRNA expression levels of caspase-3, caspase-9, and PARP. Data are presented as the means ± standard deviation (*n* = 8). ^∗^*p* < 0.05 and ^∗∗^*p* < 0.01, compared with the control group.

As demonstrated in Figures 7(a) and 7(b), the protein levels of cleaved caspase-3 were markedly increased (*p* < 0.01) in the 4 mg/kg Cu treatment group at days 21 and 42 as well as in the 8 and 16 mg/kg Cu treatment groups at day 42 in comparison to the control group. Intriguingly, the protein levels of cleaved caspase-9 in the 8 mg/kg Cu treatment group at days 21 and 42 as well as in the 4 and 16 mg/kg Cu treatment groups at day 42 were increased (*p* < 0.05 or <0.01) compared to those in the control group. Besides, the protein levels of cleaved PARP were significantly increased (*p* < 0.01) in the 8 and 16 mg/kg Cu treatment groups at days 21 and 42 when compared to the control group. In agreement with these protein expression patterns, the mRNA expression levels of caspase-3, caspase-9, and PARP were higher (*p* < 0.05 or <0.01) in the three Cu treatment groups at days 21 and 42 than in the control group (Figures 7(c) and 7(d)).

**Figure 8:**
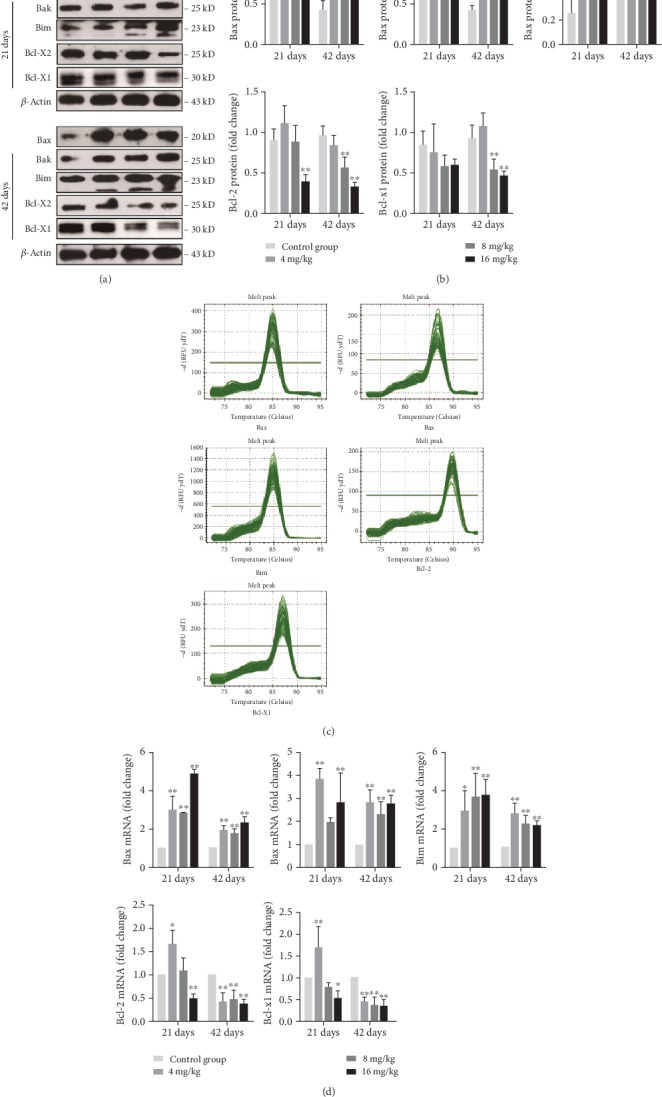
Changes of protein and mRNA expression levels of Bax, Bak, Bim, Bcl-2, and Bcl-xl in the liver at 21 and 42 days of experiment. (a) The western blot assay of Bax, Bak, Bim, Bcl-2, and Bcl-xl. (b) The relative protein expression levels of Bax, Bak, Bim, Bcl-2, and Bcl-xl. (c) The melting curve analysis of Bax, Bak, Bim, Bcl-2, and Bcl-xl. (d) The relative mRNA expression levels of Bax, Bak, Bim, Bcl-2, and Bcl-xl. Data are presented as the means ± standard deviation (*n* = 8). ^∗^*p* < 0.05 and ^∗∗^*p* < 0.01, compared with the control group.

Furthermore, when compared with the control group, the protein levels of Bax, Bak, and Bim were markedly increased (*p* < 0.01) in the three Cu treatment groups at day 42 and those of Bim were increased (*p* < 0.01) in the 8 and 16 mg/kg Cu treatment groups at day 21 (Figures 8(a) and 8(b)). On the contrary, the protein levels of Bcl-2 and Bcl-xL were markedly decreased (*p* < 0.01) in the 8 and 16 mg/kg Cu treatment groups at day 42, and those of Bcl-2 were also reduced (*p* < 0.01) in the 16 mg/kg Cu treatment group at day 21 when compared to the control group (Figures 8(a) and 8(b)). As shown in Figures 8(c) and 8(d), the mRNA expression levels of Bax and Bim in the three Cu treatment groups at days 21 and 42 were higher (*p* < 0.05 or <0.01) than those in the control group. The mRNA expression levels of Bak were significantly upregulated (*p* < 0.01) in the 4 and 16 mg/kg Cu treatment groups at days 21 and 42 as well as in the 8 mg/kg Cu treatment group at day 42. In contrast, when compared with the control group, the mRNA expression levels of Bcl-2 and Bcl-xl were lower (*p* < 0.05 or <0.01) in the three Cu treatment groups at day 42 as well as in the 16 mg/kg Cu treatment group at day 21, but higher (*p* < 0.05 or <0.01) in the 4 mg/kg Cu treatment group at day 21 (Figures 8(c) and 8(d)).

### 3.8. Changes in the Protein and mRNA Expression Levels of Apoptotic Parameters Associated with the Death Receptor Pathway in the Liver

**Figure 9:**
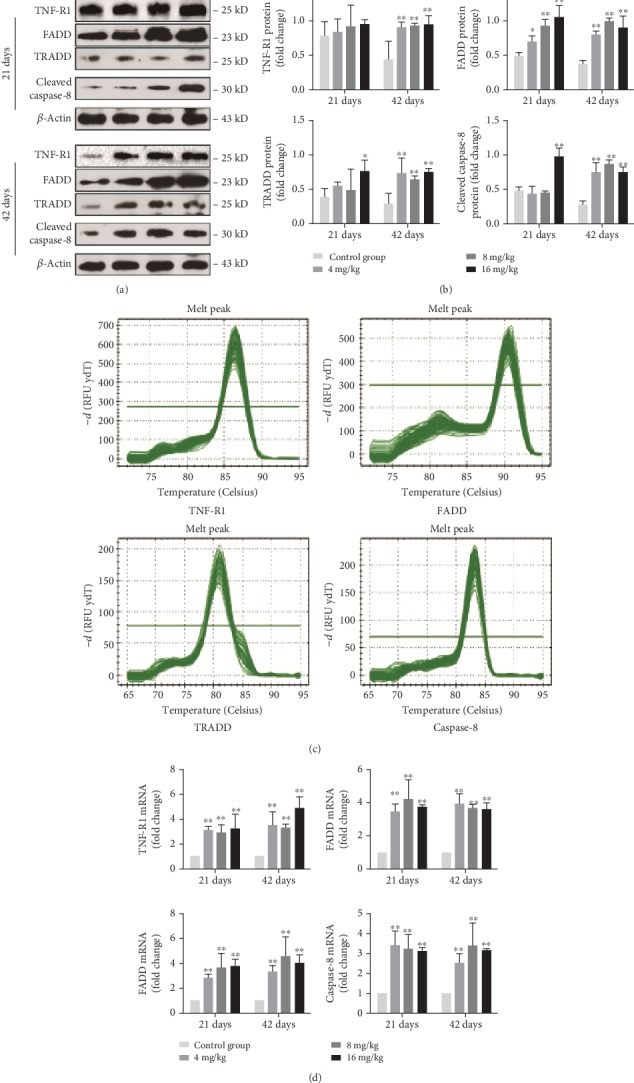
Changes of protein and mRNA expression levels of TNF-R1, FADD, TRADD, and caspase-8 in the liver at 21 and 42 days of experiment. (a) The western blot assay of TNF-R1, FADD, TRADD, and cleaved caspase-8. (b) The relative protein expression levels of TNF-R1, FADD, TRADD, and cleaved caspase-8. (c) The melting curve analysis of TNF-R1, FADD, TRADD, and caspase-8. (d) The relative mRNA expression levels of TNF-R1, FADD, TRADD, and caspase-8. Data are presented as the means ± standard deviation (*n* = 8). ^∗^*p* < 0.05 and ^∗∗^*p* < 0.01, compared with the control group.

As shown in Figures 9(a) and 9(b), the protein levels of TNF-R1 and FADD were increased (*p* < 0.05 or <0.01) in the three Cu treatment groups at day 42 and those of FADD were increased (*p* < 0.05 or <0.01) in the three Cu treatment groups at day 21, when compared to the control group. Moreover, the protein levels of TRADD and cleaved caspase-8 in the 4 and 8 mg/kg Cu treatment groups at day 42 and in the 16 mg/kg Cu treatment group at days 21 and 42 were higher (*p* < 0.05 or <0.01) than those in the control group. Similar to these protein expression data, the mRNA expression levels of TNF-R1, FADD, TRADD, and caspase-8 were significantly increased (*p* < 0.01) in the three Cu treatment groups at days 21 and 42 when compared to the control group (Figures 9(c) and 9(d)).

## 4. Discussion

Cu is required to support numerous biological activities, but it can exert cytotoxic effects when its concentration exceeds the body tolerance. Overexposure to Cu may produce some detrimental effects in the basal ganglia of human [28] and in the kidney [20], spleen, and thymus [29] in mice. Hepatocytes are regarded as one of the main target cells for Cu toxicity. Several studies have shown that Cu can induce oxidative stress, mitochondrial dysfunction, and apoptosis in hepatocytes [30, 31]. However, the underlying mechanism remains largely unclear. The present study investigated Cu-induced hepatic apoptosis and oxidative stress as well as their relationship. Additionally, our findings also indicated that Cu could induce apoptosis through the mitochondrial apoptotic and death receptor signaling pathway in the mouse liver.

To establish an animal model of Cu-induced hepatic damage, the experimental mice were treated with graded levels of Cu (0, 4, 8, and 16 mg/kg, respectively) for 42 days. The dose of Cu used in this study was based on the median lethal dose value (LD50, 245.47 mg/kg) obtained from an acute oral toxicity study. Body weight is a basic quality factor of the health state. In this study, the body weight was significantly decreased under excessive Cu exposure, indicating that excessive intake of Cu exerted a toxic effect on the mice (Figure 1(a)). Hepatic Cu concentration was markedly increased in the 16 mg/kg Cu treatment group at 42 days of the experiment (Figure 1(b)). Also, the hepatic growth index was decreased in high Cu treatment groups, suggesting that Cu overload could suppress hepatic development (Figure 1(d)). Besides, our results clearly demonstrated that the high doses of Cu could cause histopathological lesions, such as degeneration and necrosis, in the mouse liver at days 21 and 42 in a dose- and time-dependent manner.

Several mechanisms have been proposed to explain the cellular toxicity of Cu exposure. One of the most accepted is the propensity of stimulating ROS production, which results in oxidative stress [32]. In the present study, the levels of ROS were significantly increased upon Cu exposure in a time- and dose-dependent manner (Figure 3(a)). Excessive ROS production can oxidize cell components such as protein, leading to protein oxidation [33]. PC are the representative products of protein oxidation [27]. Moreover, our results clearly demonstrated that the contents of PC were increased in the three Cu treatment groups, which are consistent with the overproduction of ROS. Besides, the abilities of ASA and AHR are often used to assess the total capacity of scavenging superoxide anion and hydroxyl radical, respectively, which represent the portion of ROS [34, 35]. In the present study, the abilities of ASA and AHR were decreased in the three Cu treatment groups when compared to the control group, suggesting that the capacity of the liver to scavenge ROS products is reduced after Cu exposure. The imbalance between ROS and the antioxidant defense system can lead to oxidative stress. Thus, we further examined the effects of Cu on the activities of antioxidant enzymes and contents of nonenzymatic antioxidants in the mouse liver.

The results of this study showed that Cu decreased the activities of CAT, GSH-Px, and SOD, as well as GSH contents in the liver at different degrees (Figure 4(a)), which are in line with our earlier findings on the effects of Cu on antioxidant enzymes [5, 36]. CAT and SOD are parts of free radical-scavenging enzymes that ameliorate the damaging effects of ROS by converting them into oxygen, which is later converted into water [37]. The decreased activities of CAT and SOD can be used to indicate the accumulation of superoxide radicals and/or hydroxyl radicals in the liver. GSH, as a nonenzymatic scavenger, can scavenge a wide variety of reactive species [38]. GSH-Px works together with CAT to scavenge excess lipid peroxide and hydrogen, which is helpful for eliminating peroxide during the reaction between GSH and H_2_O_2_ [27]. Therefore, the low activities of GSH-Px are related to the accumulation of free radicals and/or depletion of GSH. To further explore the molecular basis of the changes in antioxidant enzyme activities, the mRNA expression levels of CAT, GR, GSH-Px, GST, CuZn-SOD, and MnSOD were detected in this study. The results showed that the mRNA expression levels of these antioxidant enzymes were decreased in the three Cu treatment groups at different degrees, which are consistent with the reduction of their activities. All the above results clearly indicated that Cu not only promoted ROS production but also inhibited the activities and mRNA expression of antioxidant enzymes in the liver. Thus, the imbalance between ROS production and antioxidative function could lead to oxidative stress, which in turn contributes to the occurrence of hepatic apoptosis.

It is well known that both ROS and oxidative stress can serve as the inducer of apoptosis [39]. After entering into the hepatocytes, a portion of Cu is being transported to the mitochondria for cytochrome c oxidase incorporation [40]. High levels of Cu can induce the overproduction of ROS [3], which further impairs mitochondrial electron transport and results in mitochondrial dysfunction that is linked to the occurrence of apoptosis [41]. In this study, the rates of apoptotic hepatocytes were higher in the three Cu treatment groups than in the control group. The significant depolarization of MMP was also observed in the three Cu treatment groups when compared to the control group. The changes in mitochondrial outer membrane permeabilization can cause the release of Cyt c, AIF, and Endo G from the mitochondrion into the cytosol, leading to caspase activation and apoptosis [42, 43]. Cytosolic Cyt c binds to Apaf-1 and procaspase-9 to form an apoptosome, thus resulting in caspase-9 activation [44, 45]. The activated caspase-9 cleaves and activates the downstream executioner caspase-3, which ultimately triggers PARP cleavage and apoptosis [43]. In the present study, the upregulated mRNA and protein expression levels of cytosolic Cyt c, Apaf-1, cleaved caspase-3 and caspase-9, and cleaved PARP were observed. Santos and colleagues [46] have reported that Cu induces apoptosis in Hep-G2 cells by altering the expression of caspase-3, caspase-8, caspase-9, AIF, and p53. Hosseini and coworkers [42] have also found that Cu-induced liver apoptosis is the consequence of its disruptive effect on mitochondrial membrane permeability in hepatocytes caused by Cu-induced ROS formation and Cyt c expulsion. In the present work, we also observed the increased expression levels of AIF and Endo G. It is noteworthy to mention that AIF and Endo G can translocate into the nucleus to cause DNA fragmentation and apoptosis via the caspase-independent mitochondrial pathway after MMP depolarization [47].

In addition, we found that Cu exposure increased the mRNA and protein expression levels of Bax, Bak, and Bim and decreased those of Bcl-2 and Bcl-xl. It is worth noting that Bax, Bak, and Bim belong to the proapoptotic Bcl-2 protein family. When apoptosis is initiated, Bim, as a BH3-only protein, can transfer into the mitochondrial outer membrane, promote the release of Cyt c and Endo G [48], and directly induce the conformational changes of Bax and Bak, which in turn mediate Cyt c efflux [49]. Therefore, the increased expression levels of Bim, Bax, and Bak corresponded to those of cytosolic Cyt c and Endo G in this study. Nevertheless, the antiapoptotic Bcl-2 and Bcl-xl have been reported to inhibit apoptosis by binding to Bax or Bak [50], thereby preventing the release of Cyt c and subsequent caspase activation [49]. Kawakami and colleagues [51] have found that a high intake of Cu can induce apoptosis via increasing the expression levels of Bax, Bad, caspase-3, Cyt c, and caspase-9 in PC12 cells. Chan and coworkers [52] have also observed that Cu-induced apoptosis is often accompanied by the increased expression levels of Bax and Bak as well as decreased expression level of Bcl-2 in neuroblastoma cells.

Furthermore, we found that excess Cu exposure increased the mRNA and protein expression levels of TNF-R1, FADD, TRADD, and caspase-8, which represent the important members in the death receptor signaling pathway. TNF-R1, as the receptor of TNF-*α*, plays an important role in apoptosis induction [53]. Following the combination of ligands and receptors, FADD and TRADD adaptor proteins are being recruited, which then initiate the formation of death-inducing signaling molecules and further activate caspase-8 (a central mediator of the death receptor signaling pathway) [54]. The activated caspase-8 catalyzes the proteolysis of caspase-3 and drives the cascade reactions of downstream signaling molecules [25]. All the above findings clearly revealed that the TNF-R1 signaling pathway was involved in Cu-induced hepatic apoptosis.

## 5. Conclusion

**Figure 10:**
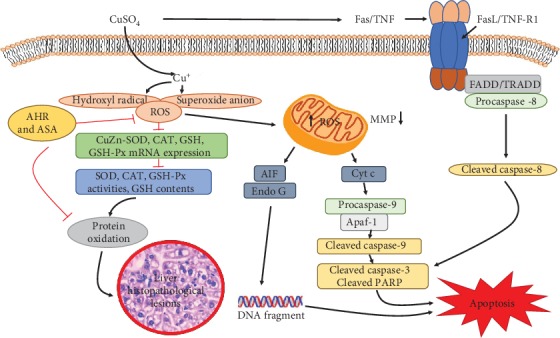


The results of this study demonstrate that exposure to excess Cu can induce hepatic oxidative damage by increasing the levels of ROS and PC, suppressing the ability to scavenge free radical and reducing the mRNA levels and activities of antioxidant enzymes, which in turn leads to hepatic lesions. The increased levels of ROS induced by Cu can impair mitochondrial membrane permeability, resulting in the activation of the mitochondrial apoptotic pathway and hepatic apoptosis. Additionally, it has been found that the TNF-R1 signaling pathway is involved in Cu-induced apoptotic cell death in the mouse liver (Figure 10).

## Figures and Tables

**Table 1:** Primer sequences of genes selected for analysis by qRT-PCR.

Target gene	Accession number	Primer	Primer sequence (5′–3′)	Product size	Tm (°C)
CAT	NM-009804	Forward	CCTATTGCCGTTCGATTCTC	119 bp	58.8

		Reverse	CCCACAAGATCCCAGTTACC		
GR	NM_010344	Forward	AAGCGCTTCTCACCCCAGTT	121 bp	60.9
		Reverse	GGGTGGCTGAAGACCACAGTA		

GSH-Px	NM_008160	Forward	TACACCGAGATGAACGATCTG	102 bp	56.9

		Reverse	ATTCTTGCCATTCTCCTGGT		
GST	NM_019946	Forward	GGGATTGGCTGTGATGAGAT	121 bp	57.2
		Reverse	AGGTAGGATGAATGGCAACTG		

CuZn-SOD	NM_011434	Forward	GGGTTCCACGTCCATCAGTA	113 bp	61

		Reverse	CAGGTCTCCAACATGCCTCT		
MnSOD	NM_013671	Forward	AACTCAGGTCGCTCTTCAGC	113 bp	61
		Reverse	CTCCAGCAACTCTCCTTTGG		

Cyt c	NM_025567	Forward	GGCTCCTCCCATCTACACAG	117 bp	56.4

		Reverse	TCATGCTCTGGTTCTGATGC		
Endo G	NM_007931	Forward	TCGAGCTACGTTCCTACGTG	119 bp	56.2
		Reverse	ATTGGGCACGAAGAGCAATC		

AIF	NM_012019	Forward	GATCAGGGCACCAAGTCACG	146 bp	58.4

		Reverse	GAGGTCGCATGTATGGCAGT		
Apaf-1	NM_009684	Forward	GCAAGGACACAGATGGTGGA	120 bp	56.6
		Reverse	TCTGCTGAATCGCATGAACC		

Bax	NM_007527	Forward	ATGCGTCCACCAAGAAGC	163 bp	59.8

		Reverse	CAGTTGAAGTTGCCATCAGC		
Bak	NM_007523	Forward	CGCTACGACACAGAGTTCCA	175 bp	57.7
		Reverse	CACGCTGGTAGACGTACAGG		

Bim	NM_207680	Forward	CCTTCTGATGTAAGTTCTGAGTGTG	113 bp	57.6

		Reverse	CCTTGCGGTTCTGTCTGTAG		
Bcl-2	NM_009741	Forward	AGCCTGAGAGCAACCCAAT	159 bp	58.7
		Reverse	AGCGACGAGAGAAGTCATCC		

Bcl-xl	NM_009743	Forward	TGTGGATCTCTACGGGAACA	117 bp	56.4

		Reverse	AAGAGTGAGCCCAGCAGAAC		
TNF-R1	NM_011609	Forward	AATGCAGACCTTGCGATTCT	114 bp	57.8
		Reverse	CATCTCCAGCCTCTCGATCT		

FADD	NM_010175	Forward	CGTGAGAAACGAAAGCTGG	142 bp	58.7

		Reverse	CTGCAGTAGATCGTGTCGGC		
TRADD	NM_1033161	Forward	TGGCTGACTGATGAAGAGCG	112 bp	59.2
		Reverse	CACACGTCAGTTTGCAGAGC		

Caspase-3	NM_009810	Forward	ACATGGGAGCAAGTCAGTGG	149 bp	58.3

		Reverse	CGTCCACATCCGTACCAGAG		
Caspase-9	NM_015733	Forward	GAGGTGAAGAACGACCTGAC	103 bp	58.8
		Reverse	AGAGGATGACCACCACAAAG		

Caspase-8	NM_009812	Forward	GCTGCCCTCAAGTTCCTGT	118 bp	60.9

		Reverse	GATTGCCTTCCTCCAACATC		
PARP	NM_007415	Forward	CTCTCCAATCGCTTCTACAC	108 bp	57.8
		Reverse	GTTGTCTAGCATCTCCACCT		

*β*-Actin	NM_007393	Forward	GCTGTGCTATGTTGCTCTAG	117 bp	60.9
		Reverse	CGCTCGTTGCCAATAGTG		

## Data Availability

The data used to support the findings of this study are included within the article.
